# Supplementary material for: Planctomycetes of the Genus Singulisphaera Possess Chitinolytic Capabilities
Source: Microorganisms. 2024 Jun 22;12(7):1266. doi: 10.3390/microorganisms12071266 (PMC11279305; doi:10.3390/microorganisms12071266)
Supplement: Supplementary file 1 [file microorganisms-12-01266-s001.zip › microorganisms-3057152-supplementary.pdf]

**Supplementary materials**

# Planctomycetes of the genus *Singulisphaera* possess chitinolytic capabilities

**Anastasia A. Ivanova [1*], Daniil G. Naumoff [1*], Irina S. Kulichevskaya [1], Andrey L. Rakitin[2], Andrey V. Mardanov [2], Nikolai V. Ravin [2], Svetlana N. Dedysh [1**]**

[1] *Winogradsky Institute of Microbiology, Research Center of Biotechnology of the Russian Academy of Sciences, Moscow 119071, Russia*
[2] *Institute of Bioengineering, Research Center of Biotechnology of the Russian Academy of Sciences, Moscow 119071, Russia*
* *These authors have contributed equally to this work.*
** *Correspondence: dedysh@mail.ru*

## TABLE OF CONTENTS

**Sequences** .......................................................................................................................... 3

Three putative chitinases revealed by dbCAN3-based search in the genome of *Singulisphaera* sp. Ch08.

**Supplementary Figure S1** ........................................................................................................ 4

Effects of pH and temperature on the chitobiosidase activity of the recombinant ChiA chitinase.

**Three putative chitinases revealed by dbCAN3-based search.**

>**ChiA** chitinase, GH18 family (8966861 - 8967991) [*Singulisphaera* sp. strain Ch08]
MKHFLTRRSLLWAAVLAGFSSPAATLAAPDQNPTDKVFVGYLFGQPRNINFGLYTHLCHA
FVTADAEGNLNRSRSVPNRDLINDAHKAGVKVLVSLGGWGWDDQFAAIVSKPEAEERYAK
AVMDLVEKSNYDGIDFDWEYPDNKQEVVGFERLSRRFRKDLDAIGARNGRSMLLTMAATS
HPGTLRWLDKTFLLETMDWVNVMTYDFTGDWTNYAGHHSPLYASSKQPKGNSHSTESTMK
YLVEERGLPANRLAVGVPLYGRGFAVSEPYASTKGIAKNRIPQGEYSNLHKLLNAQGWTR
HWDDETKNPWLFAPDRSMVMGYDDAESIAIKTDWLLKQGFRGIFFWQIAGDRLPDGSNPL
QEAARKKWAAGARPSGK

>**ChiB** chitinase, GH18 family (8423162 - 8425381) [*Singulisphaera* sp. strain Ch08]
MTGAWTSGFQAQMSLNNSQPTSVADWRLEFDLDAKITSIWDATIVSQTGNHYVIAGAAWD
KTIPAGGVVSFGFVANASHSAAPSHEVLNGIPLGTPLPVVPGLSISDASVAEGNTGTTDL
TFVVNLSEPAATAVAVSYTTSDGTAKGGSDYQTSHGTLNFAPGETRKTINVRVNGDLLVE
PDETLIVSLVSPTGATLVRDRATGTIRNDDTPPLPPANGDVEFKVTSDWVSGFNGEVVVH
NRVTTPMNDWTLEFDFAGQITSLWNGVLVNRNGNHVVVKGADWNKTIAPGSSVSFGFTAS
PGGNGAGLTNFVLQGQSTGGSGGGGATNHAPVAVADSAFTSAGQPVPINVLSNDSDPDRD
PISLTSFTQSQNGTVSVNAAGTLTYTPKPGFTGLDSFSYIVSDGRGGTDTASVAVTVSAP
VAPSIWPAQFYAPYVDMGLYPTYNLAAAAQSTGVKYFTLAFIVADPQNQPSWGGYSEYAV
NGGEFDLNMRSQVAAVRALGGDVMASFGGASGRELAQAITDVNALTAAYQKVIDAYGLTH
LDFDIEGAASADLASIHRRSQAIANLQANATAAGKTLNVWFTLPVLPTGLTVDGVNLLQS
ALRYGVRIDGVNIMTMDYGDGAAPNPRGQMGEYAIDAANSLFRQLQTLYGSSRTSAQLWS
MIGVTPMIGLNDVTTETFDQEDAAQLTAFAEQHGMGRISIWSLNRDKQAPSGALSYVDSG
SSSLVQQPFEFSKIFLPYQG

>**CelA** cellulase, GH5 family (1537669 – 1540458) [*Singulisphaera* sp. strain Ch08]
MIKDAGYNATITSGQTTSFGFNGAPGHVTAPPANYQLNGVPLDGSGPSPVQSPPTVATPA
SAASSSVTGKTVALAVLGADDGGESNLTYRWTTVGTSPAPVAFSTNGSNAAKNTIATFSK
TGAYTFQVSIADAGGRSITSNVNVTVAQTLTAVVVSPATVTVAANATQSFSAQGLDQLGS
LLATQPPFSWSIASGAGTINGSGMYTAPSSAASATVRAASAGISGTSSVTVNPPVVPGNA
TASAVYSVASDWGSGFTGNIALTNTGSSPINGWTLEFDFPATITQIWNAKIVSHVGNHYV
IQDLGYNAKLAPGQSTSFGFNGQPGGNAGTPARYILNGVSLGGGASVLPALSVGDVTVSV
GNTATQALFTVTLSQPATAPISVAYTTVDGTAKAGTDYHAVSGFLNFSAGQTTQTLAVPV
FAMTVANKPDLKFSLVLSNPNGASLSRTTAIGTIHDIYTSPATSPTIRINNVTVTAPSGQ
TSSGFFQTAGNQIVNREGTAVKIAGINWFGFETGNYVPHGLWARNYQDMMNQMKQLGFNT
IRMPFSNAIFNPANVPNSINFGLNSDLQGLSSLQILDKIVSYAGQIGLRIILDHHSALPD
NHANETLWYVPGDANNSEQVWINNWVALAQRYAGNPTVIGADLKNEPHGQASWGDGNLAT
DWRLAAQRAGNAVLAANPNWLIFVEGIETYNGQSTWWGGNLMGARQHPVVLNVANRVVYS
PHDYPASVYNQSWFNAANYPNNLTSVWNQYWGYLYQENIAPIWLGEFGSKLQTTSDQQWY
QQITSYLGNTTSSSSVPGQQGMSWTWWSWNPNSRDTGGILQDDWKTASSTKVQGLVPIQF
AFPTTSGPGTGTATATFVVTLSAPSTQTVTVAYSTANGTAASGTNYVATNGTLTFAPGQT
QATVSVTVLYDPTLTRDATFSILLANPTNSTLDGTGRGTGTIRAGT

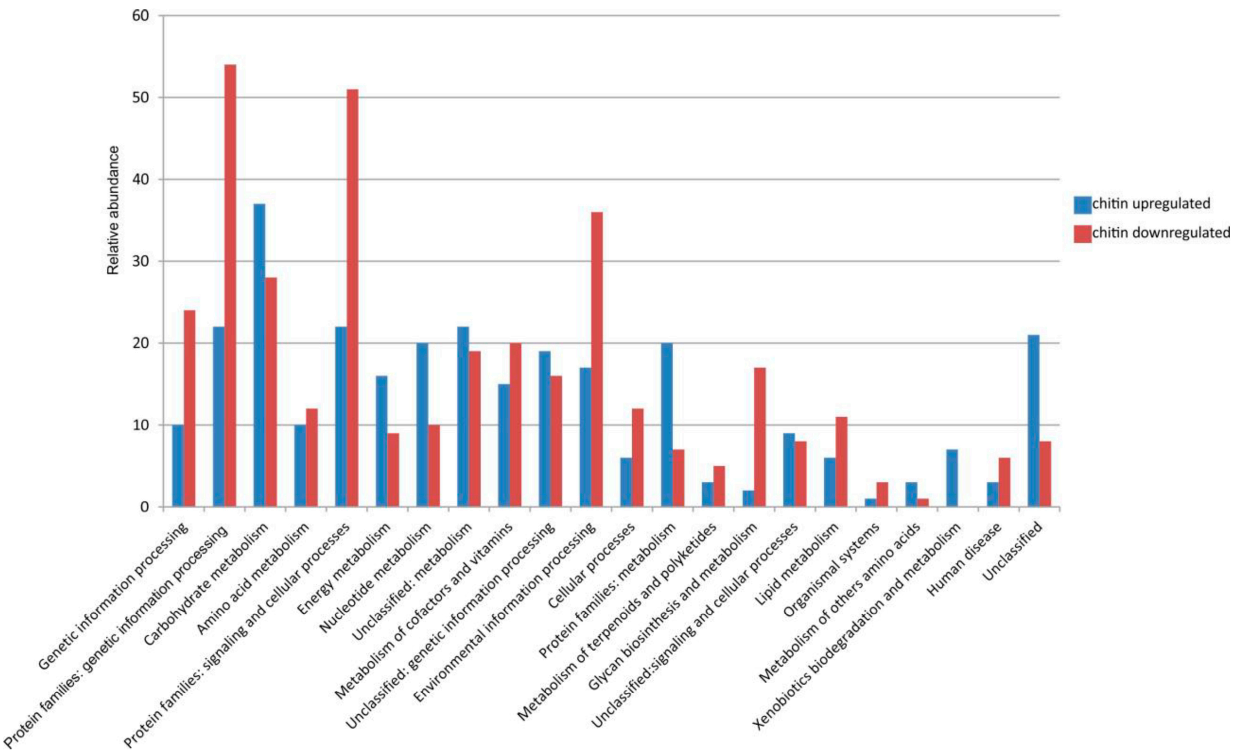


**Figure S1**. Up-regulation (blue) and down-regulation (red) of genes distributed to various KEGG categories in strain Ch08 during growth on chitin. Transcripts with fold change ≥ 2 were taken in the analysis.

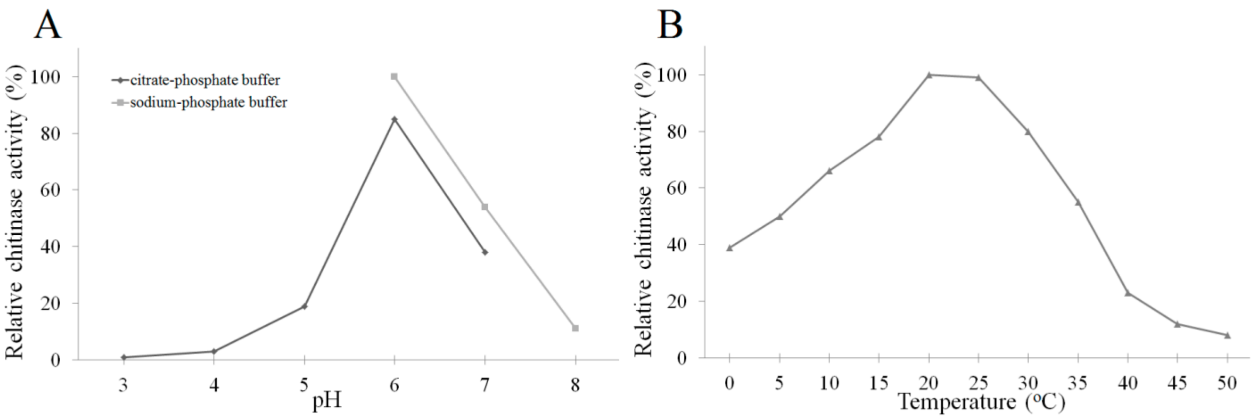


**Figure S2.** Effects of pH (A) and temperature (B) on the chitobiosidase activity of the recombinant ChiA chitinase.
